# Rational Design of Metal-Free Nitrogen-Doped Carbon for Controllable Reduction of CO_2_ to Syngas

**DOI:** 10.3390/molecules30040953

**Published:** 2025-02-18

**Authors:** Guangbin An, Kang Wang, Min Yang, Jiye Zhang, Haijian Zhong, Liang Wang, Huazhang Guo

**Affiliations:** 1 Institute of Nanochemistry and Nanobiology, School of Environmental and Chemical Engineering, Shanghai University, 99 Shangda Road, Shanghai 200444, China; 2020agb@shu.edu.cn (G.A.); kangwang@shu.edu.cn (K.W.); wangl@shu.edu.cn (L.W.); 2 School of Information Engineering, Gannan Medical University, Ganzhou 341000, China; min_yang100@163.com; 3 School of Materials Science and Engineering, Shanghai University, 99 Shangda Road, Shanghai 200444, China; jychang@shu.edu.cn

**Keywords:** nitrogen-doped carbon, carbon-based catalyst, electrocatalytic, CO/H_2_ syngas

## Abstract

The electrocatalytic reduction of CO_2_ (ECO_2_RR) to syngas with tunable CO/H_2_ ratios offers a promising route for sustainable energy conversion and chemical production. Here, we report a series of N-doped carbon black (NCBx) catalysts with tailored nitrogen species that enable precise control over the composition of syngas. Among the catalysts, NCB3 exhibits the optimal performance, achieving high CO selectivity (64.14%) and activity (1.9 mA cm^−2^) in an H-type cell at −0.9 V. Furthermore, NCB3 produces syngas with a wide range of CO/H_2_ ratios (0.52 to 4.77) across the applied potentials (−0.5 to −1.0 V). Stability tests confirm the robust durability of NCB3, which maintains consistent activity and selectivity over prolonged electrolysis. This work demonstrates the critical role of nitrogen species in tuning ECO_2_RR pathways and establishes a strategy for designing efficient and stable carbon-based catalysts for CO_2_ utilization and syngas production.

## 1. Introduction

Syngas, a mixture of H_2_ and CO, serves as a vital industrial feedstock for producing diverse hydrocarbons via Fischer–Tropsch synthesis [1,2,3,4]. Currently, the predominant method used for large-scale syngas production involves steam reforming, which utilizes fossil fuels such as coal, petroleum coke, and natural gas as raw materials. By adjusting temperature and pressure, this process achieves varying syngas compositions [5,6]. However, traditional steam reforming has significant drawbacks: it is highly energy-intensive, relies heavily on fossil fuels, and necessitates frequent operational adjustments to optimize syngas ratios [7]. These factors not only deplete finite fossil fuel resources and intensify the global energy crisis but also result in excessive CO_2_ emissions, contributing to severe environmental challenges. Due to the urgent need for carbon neutrality, developing a sustainable and eco-friendly pathway for syngas production is imperative.

The electrochemical CO_2_ reduction reaction (ECO_2_RR) can produce syngas through the two-electron (2e^−^) pathway of ECO_2_RR combined with hydrogen evolution (HER) [8,9,10,11]. This approach not only mitigates the environmental impact of CO_2_ emissions but also offers a sustainable alternative to conventional syngas production methods, achieving the dual benefits of addressing carbon emissions and advancing industrial processes [12,13]. Currently, transition metal-based catalysts are considered excellent candidates for electrocatalytic CO production. However, their inherent ability to suppress HER limits their capacity to produce syngas with tunable H_2_/CO ratios [3,8,14]. Consequently, there is an urgent need to develop innovative electrocatalysts with abundant active sites and robust efficiency that are capable of precisely controlling the H_2_/CO ratio over a wide potential range to unlock the full potential of this sustainable pathway. Recent studies have highlighted the potential of metal-free nitrogen (N)-doped carbon materials, such as N-doped carbon nanosheets [15,16,17], N-doped carbon nanofibers [18,19,20], and N-doped graphene foams [21,22,23]. These materials have gained significant attention due to their abundance, high defect density, excellent conductivity, and remarkable chemical stability. These features not only promote CO_2_ adsorption but also address the limitations of transition metal-based catalysts, enabling the production of syngas with a tunable composition at controllable working potentials.

Here, we present N-doped carbon black (NCB) as an efficient and robust catalyst for the electrochemical conversion of CO_2_ to syngas. Unlike conventional metal-free carbon-based catalysts, the NCB catalyst exhibits a broad syngas ratio, ranging from 0.52 to 4.77, and exceptional long-term stability, maintaining consistent performance over 25 h of continuous electrolysis without any loss in current density. Notably, the NCB catalyst demonstrates a symmetric syngas ratio performance across a potential range of −0.5 to −1.0 V, enabling the continuous production of tunable syngas under fluctuating renewable energy conditions. This unique property allows the NCB catalyst to produce syngas with similar compositions at both high and low operating potentials, with the syngas ratio consistently falling within the ideal range for practical applications.

## 2. Results and Discussion

### 2.1. Morphology Characterization of NCB Catalysts

Carbon black (CB) was chosen as the base material for our study due to its high surface area, low cost, and scalability for large-scale production. Additionally, its nanoparticulate form enhances the diffusion pathways for CO_2_, ensuring a sufficiently high CO_2_ concentration at elevated current densities. However, the limited surface defects in pure CB restrict its catalytic activity. To address this, we introduced chitosan—a nitrogen-rich biopolymer with abundant amino functional groups—as an N-dopant to improve the electrocatalytic performance of CB.

**Figure 1:**
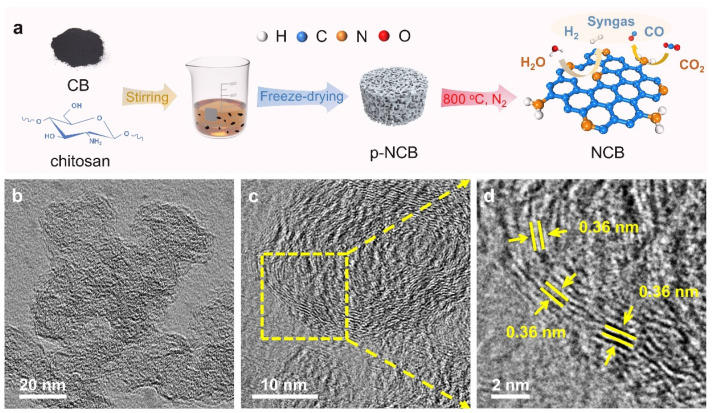
(**a**) Is a schematic of the synthesis process for NCB catalysts. (**b**) is a TEM image of NCB3. (**c**,**d**) are HRTEM images of NCB3.

The process of synthesizing NCB catalysts with varying nitrogen contents (denoted as NCBx, x = 1, 2, 3, 4) is illustrated in Figure 1a. NCBx was prepared by mixing chitosan and carbon black in solution, followed by freeze-drying and high-temperature calcination. As shown in Table S1, the process achieved a maximum yield of 74.41% for NCB3, underscoring its simplicity and scalability. Freeze-dried precursor samples (p-NCB) exhibited loose, porous structures with visible pores (Figure S1a–d), likely due to the low mass concentration of chitosan and the sublimation of water molecules during freeze-drying, which created a porous network within the chitosan’s molecular chains [24].

**Figure 2:**
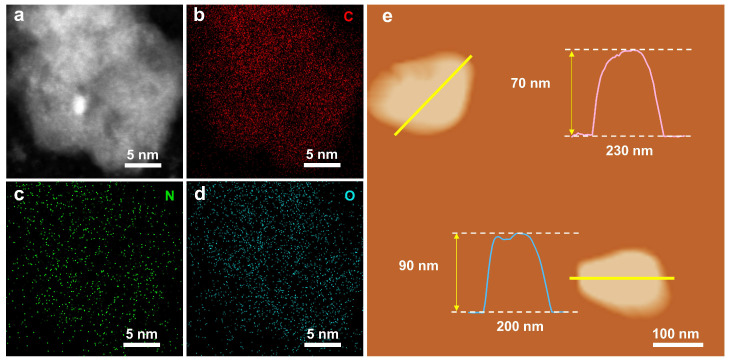
(**a**–**d**) HAADF-STEM and elemental mapping images of NCB3. (**e**) AFM image of NCB3.

Transmission electron microscopy (TEM) images (Figure 1b and Figure S1e–h) confirmed the nanoparticulate morphology of the NCBx catalysts. The high-resolution TEM (HRTEM) analysis of NCB3 (Figure 1c,d) revealed an amorphous carbon structure with a lattice spacing of approximately 0.36 nm. High-angle annular dark-field scanning transmission electron microscopy (HAADF-STEM, JEOL, JEM-2100Plus, Tokyo, Japan) images further confirmed the disordered structure of NCB3 (Figure 2a). Energy-dispersive X-ray spectroscopy (EDS) mapping demonstrated a uniform distribution of N and O within the carbon matrix (Figure 2b–d). Atomic force microscopy (AFM) analysis (Figure 2e) revealed that the height of NCB3 was about 70–90 nm.

### 2.2. Structure Characterization of NCB Catalysts

**Figure 3:**
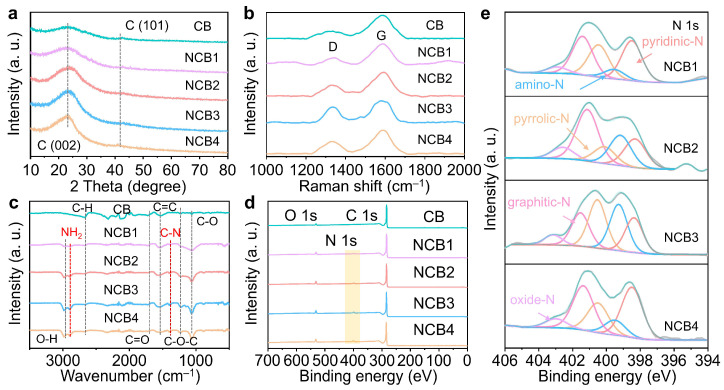
(**a**) XRD patterns, (**b**) Raman spectra, and (**c**) FT-IR spectra of CB, NCB1, NCB2, NCB3, and NCB4. (**d**) XPS survey spectra and (**e**) the high-resolution N 1s XPS spectra of NCB1, NCB2, NCB3, and NCB4.

A comprehensive suite of advanced characterization methods was employed to elucidate the structural distinctions of the NCBx catalysts. Their X-ray diffraction (XRD) patterns (Figure 3a) reveal a broad diffraction peak around 23°, corresponding to the (002) planes of carbon. Notably, the peak position remains invariant with increasing nitrogen content, indicating that nitrogen doping does not alter the interlayer spacing of carbon. Based on Bragg’s equation [25], the lattice spacing of the (002) planes for NCB3 is approximately 0.36 nm, consistent with the TEM results and slightly larger than that of graphite (0.34 nm). This expansion is likely due to the incorporation of nitrogen atoms into the carbon framework. Raman spectroscopy provided insights into the defect degree of the carbon nanomaterials. As shown in Figure 3b, the NCBx catalysts exhibit two characteristic peaks: the D-band at ~1345 cm^−1^ (defects) and the G-band at ~1583 cm^−1^ (graphitic sp^2^ domains). The I_D_/I_G_ ratio, indicative of defect density, is higher for NCB3 (0.78) than NCB1 (0.31), NCB2 (0.45), and NCB4 (0.53), demonstrating that N-doping significantly enhances defect formation, with NCB3 exhibiting the most pronounced defect structure.

Fourier-transform infrared spectroscopy (FT-IR) analysis (Figure 3c) confirms the functional groups present in the NCBx catalysts. Peaks associated with O-H (~2986 cm^−1^), C-H (~2668 and 889 cm^−1^), C=O (~1722 cm^−1^), C=C (~1562 cm^−1^), C-O-C (~1216 cm^−1^), and C-O (~1067 cm^−1^) can be observed in all samples [26]. Importantly, peaks corresponding to -NH_2_ (~2895 and 574 cm^−1^) and C-N (~2104 cm^−1^) stretching further confirm the incorporation of nitrogen [26]. The intensity of the -NH_2_ peaks is highest for NCB3, suggesting it possesses the most abundant amino functional groups among the samples. X-ray photoelectron spectroscopy (XPS) provided detailed insights into their elemental composition and surface chemical states. As shown in Figure 3d and Table S2, all NCBx samples contain C, N, and O, with the nitrogen content increasing from 3.14 at% for NCB1 to 5.28 at% for NCB4. Deconvoluted C 1s spectra (Figure S2 and Table S3) revealed peaks corresponding to C-C/C=C (284.8 eV), C-O (286.1 eV), C-N (287.4 eV), and C=O (288.3 eV) bonds, with C-N configurations confirming nitrogen doping. C=O (531.8 eV) and C-O (533.1 eV) were found in the O 1s spectra (Figure S3 and Table S4). The N 1s spectra (Figure 3e and Table S5) identified five types of nitrogen species: pyridine N (398.5 eV), amino N (399.5 eV), pyrrole N (400.5 eV), graphitic N (401.4 eV), and oxidized N (402.91 eV) [16]. Interestingly, the relative abundances of nitrogen species vary with the chitosan content. NCB3 has the highest amino N content (26.94 at%) and the lowest pyridine N content (20.30 at%), consistent with the FT-IR results. This suggests that the amino groups from chitosan preferentially occupy nitrogen sites, which play a critical role in CO and H_2_ production during CO_2_ reduction. The minimal graphitic N content in NCB3 aligns with its highly defective structure, observed via Raman spectroscopy.

**Figure 4:**
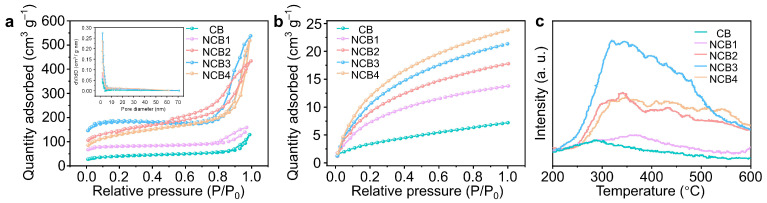
(**a**) N_2_ adsorption–desorption isotherm (inset: related pore size distribution), (**b**) CO_2_ adsorption isotherms, and (**c**) CO_2_-TPD of CB, NCB1, NCB2, NCB3, and NCB4.

The pore structure and surface area of the NCBx catalysts were evaluated through nitrogen adsorption–desorption isotherms (Figure 4a and Table S6). All samples exhibited type IV isotherms with hysteresis loops, characteristic of mesoporous materials. Their specific surface area increases with the chitosan content, reaching a maximum in NCB3 (602.71 m^2^ g^−1^), followed by a slight decrease in NCB4 (582.70 m^2^ g^−1^). Their corresponding pore size distribution confirms that NCBx catalysts have narrow mesopores, with NCB3 displaying the largest total pore volume, facilitating high-density active site exposure and the improved diffusion of reactants [27]. The CO_2_ adsorption capacities of the catalysts were also examined (Figure 4b). At 1 atm, the adsorption capacities of NCB1, NCB2, NCB3, and NCB4 were 7.4, 13.1, 21.2, and 17.5 cm^3^ g^−1^, respectively, with NCB3 exhibiting the highest adsorption capacity. This enhanced adsorption is attributed to its optimized pore structure and nitrogen functionalities.

Temperature-programmed desorption (TPD) experiments were conducted to evaluate the samples’ CO_2_ chemisorption behavior (Figure 4c). All catalysts exhibit broad desorption peaks between 250 and 600 °C, with NCB3 showing the highest peak intensity at ~350 °C, indicative of moderate-strength CO_2_ adsorption sites. This suggests that NCB3 has the most effective surface sites for CO_2_ adsorption and activation. Notably, CO_2_-TPD revealed characteristic peaks corresponding to Lewis base sites, while NH_3_-TPD (Figure S4) showed no significant signals, indicating that NCBx catalysts are of a predominantly Lewis base character [28].

### 2.3. Electrocatalytic Activity Tests of NCB Catalysts

**Figure 5:**
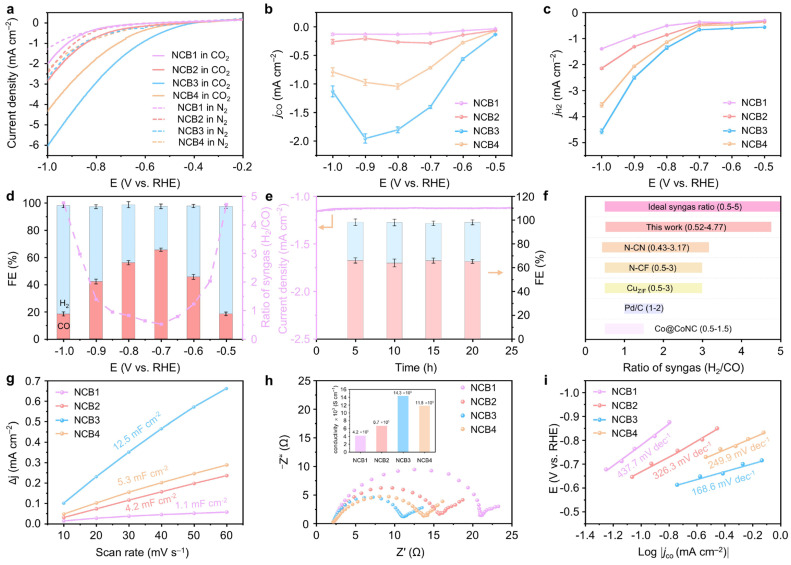
(**a**) LSV curves in CO_2_/N_2_-saturated 0.1 M KHCO_3_ solution. (**b**) CO and (**c**) and H_2_ partial current density of NCB1, NCB2, NCB3 and NCB4. (**d**) FE of CO and H_2_ and mole ratio of H_2_/CO in NCB3. (**e**) The long-term stability and the corresponding FE of CO and H_2_ at −0.7 V vs. the RHE of NCB3 for 25 h. (**f**) Syngas ratio of different catalysts. (**g**) The measured double-layer capacitance, (**h**) the Nyquist plots of the CO_2_-saturated 0.1 M KHCO_3_ electrolyte, and (**i**) a Tafel plot of the CO production of NCB1, NCB2, NCB3, and NCB4.

ECO_2_RR primarily occurs at the gas–solid–liquid three-phase interface, where enhanced catalyst hydrophilicity promotes CO_2_ diffusion, thus improving catalytic activity [29]. To investigate the interfacial hydrophilicity of the catalysts during ECO_2_RR, the contact angle of a CO_2_-saturated 0.1 M KHCO_3_ solution on the catalyst surface was measured prior to the electrocatalytic reaction. As depicted in Figure S5, NCB1 exhibited a contact angle of 128.73°, and the hydrophilicity of the catalysts progressively improved with increasing nitrogen contents. These results indicate that enhanced hydrophilicity facilitates the formation of surface-bound CO* intermediates, thereby boosting CO selectivity during the ECO_2_RR [30]. The electrocatalytic CO_2_ reduction performance of the NCBx catalysts was investigated in an H-type cell, as shown in Figure 5 and Figure S6. Linear sweep voltammetry (LSV) curves (Figure 5a) reveal higher current densities for all NCBx catalysts in CO_2_-saturated electrolytes compared to N_2_-saturated conditions, highlighting their ECO_2_RR activity [31]. Among the catalysts, NCB3 exhibited the lowest onset potential (−0.4 V) and the highest current density (6 mA cm^−2^ at −0.4 V), outperforming NCB1 (−0.8 V, 2 mA cm^−2^), NCB2 (−0.73 V, 2.7 mA cm^−2^), and NCB4 (−0.62 V, 4.3 mA cm^−2^). Notably, pure CB displayed the poorest performance (Figure S7), underscoring the efficacy of nitrogen doping in enhancing catalytic activity.

Potentiostatic electrolysis further confirmed the performance of the catalysts, revealing no liquid-phase products. As shown in Figure 5b–c and Figure S8, NCB3 achieved the highest total current density (5.2 mA cm^−2^ at −1.0 V) and the maximum partial current densities for CO (j_CO_ = 1.9 mA cm^−2^) and H_2_ (j_H2_ = 2.6 mA cm^−2^) at −0.9 V. Interestingly, j_CO_ increased with the potential up to −0.9 V, and then decreased beyond that point, while j_H2_ showed a steady rise, accelerating after −0.7 V. This trend suggests that ECO_2_RR dominates at lower potentials, favoring CO production, while HER becomes predominant at higher potentials due to its lower energy barrier near the water decomposition potential.

The Faradaic efficiencies (FEs) of the gas products are presented in Figure 5d and Figure S9. For NCB3, FE_CO_ reached a maximum of 64.14% at −0.7 V, which declined as the potential increased. Pure CB exhibited a negligible FE_CO_ (Figure S10), validating the importance of nitrogen doping for regulating CO and H_2_ production. Control experiments with alternating CO_2_ and Ar feed gasses (Figure S11) confirmed that the CO and H_2_ products originated solely from CO_2_ reduction, as no detectable gas-phase products were observed under Ar. NCB3 demonstrated a tunable syngas ratio across a wide potential range (−0.5 to −1.0 V), with an ideal ratio (CO/H_2_ = 0.52–4.77) suitable for various renewable energy applications (Figure 5d). This symmetric distribution of syngas was unique to NCB3 among the NCBx series (Figure S12). Additionally, NCB3 maintained a stable performance over 25 h of continuous operation (Figure 5e), with a constant syngas ratio (≈0.47), highlighting its durability and scalability. Figure 5f and Table S7 compare the syngas ratio range of NCB3 with other reported catalysts [5,8,15,32,33,34,35,36,37,38,39,40], demonstrating its competitive performance and cost-effective preparation.

Electrochemical active surface area (ECSA) measurements via double-layer capacitance (Cdl) showed that NCB3 (12.5 mF cm^−2^) had the highest ECSA among the catalysts studied (Figure 5g and Figure S13), consistent with its superior ECO_2_RR activity. The enhanced performance of NCB3 was further corroborated by electrochemical impedance spectroscopy (EIS) and Tafel slope analyses. As shown in Figure 5h, NCB3 exhibited the lowest charge transfer resistance, facilitating efficient CO_2_ adsorption and activation during the first reaction step. Conductivity measurements using a four-point probe (inset of Figure 5h, Table S8) confirmed the superior conductivity of NCB3, while its minimal Tafel slope (Figure 5i) indicated excellent reaction kinetics [35]. These results collectively establish NCB3 as a highly efficient and tunable electrocatalyst for the reduction of CO_2_ to syngas, offering significant promise for industrial applications [27].

The overall atomic content of N plays a pivotal role in determining the overall ECO_2_RR performance of a catalyst, yet it alone is unable to fully explain their activity and selectivity toward specific products. Based on our previous research [26], we hypothesize that specific N species serve as active sites with preferential selectivity for specific products in H-type cells and flow cells. Notably, amino N and pyridinic N play a dominant role in the generation of CO and H_2_. To validate this hypothesis, we analyzed the FEs of CO and H_2_ produced by the NCBx catalysts at −0.9 V and correlated these values with their contents of different types of N species, as determined by the N 1s XPS analysis data (Figures S14 and S15). As the total N content increases, from NCB1 to NCB4, the content of amino N shows a positive volcanic-type trend of increasing first and then decreasing. A similar trend is observed in CO selectivity, suggesting a direct correlation. Among the catalysts, NCB3 exhibits the highest content of amino N (26.9%), leading to a corresponding selectivity for CO of 42.1% and 44.5% in H-type and flow cells, respectively (Figure S14a). In contrast, pyridinic N follows an inverse volcanic-type trend, first decreasing and then increasing with higher total N contents. Similarly, its selectivity for H_2_ also exhibits the same trend. NCB3 has the lowest content of pyridinic N (20.3%), exhibiting a corresponding selectivity for H_2_ of 55.1% in H-type cells and 53.5% in flow cells (Figure S14b). This correlation remains consistent across all tested potentials except −0.9 V. In contrast, other nitrogen configurations show no significant linear correlation with CO and H_2_ selectivity (Figure S15). These findings strongly suggest that amino N plays a dominant role in CO production, while pyridinic N primarily governs H_2_ evolution, offering new insights into the rational design of metal-free N-doped carbon catalysts for tunable syngas generation.

## 3. Materials and Methods

### 3.1. Materials

Chitosan, acetic acid, potassium bicarbonate, Nafion 117 solution (5 wt%), and isopropanol were purchased from Sigma-Aldrich (St. Louis, CA, USA). Carbon black (Cabot, BP2000) was purchased from Suzhou Yilongsheng Energy Technology Co., Ltd., Suzhou, China. The CO_2_ and Ar (99.999%) were provided by the Nanjing special gas company, (Najin, China). Unless otherwise specified, all the materials were used as received without further purification.

### 3.2. Synthesis of NCB Catalysts

Firstly, 0.15 g of carbon black was dissolved in 10 mL of acetic acid solution (0.1 mol/L) and stirred ultrasonically for 0.5 h to obtain solution A. Then, chitosan (0.15 g, 0.3 g, 0.45 g, or 0.6 g) was dissolved in 20 mL of acetic acid solution (0.1 mol/L), with ultrasonic stirring carried out for 0.5 h to fully dissolve it, and solution B was obtained. Then, solution A was added to the series of solution B, respectively, and ultrasonic stirring was performed for 1 h to make sure the mixture was fully mixed and uniform, and solution C was obtained. Secondly, the precursor NCBx (p-NCBx, x = 1, 2, 3, and 4) was obtained from solution C by a freeze-drying process. Finally, the ground solid powder was placed in a tubular furnace and raised to 800 °C for 2 h at a heating rate of 5 °C min^−1^ under a N_2_ atmosphere. When its temperature dropped to room temperature, the solid powder was washed with water and ethanol several times and then dried in an oven at 60 °C to obtain NCBx.

### 3.3. Preparation of Working Electrode

A total of 2 mg of NCBx powder was ultrasonically dispersed in 800 μL of isopropyl alcohol, 150 μL of deionized water, and 50 μL of Nafion perfluorinated resin solution (5 wt%) for 1 h to form a homogeneous catalyst ink. Then, this ink was applied to hydrophobic carbon paper (Toray carbon paper with an active area of 1 × 1 cm^2^) using multiple 20 μL pipette drops, so that each electrode contained 100 μL of ink. Finally, the prepared working electrode was dried at room temperature for 12 h.

### 3.4. Electrochemical Test of CO_2_ Reduction Reaction

The electrochemical experiments were carried out in a gas-tight H-cell (Figure S6) containing 30 mL of electrolyte, which was split between two single cells and separated by a proton exchange membrane (Nafion 117). A Ag/AgCl (filled with saturated KCl solution) electrode and Pt sheet electrode were used as the reference electrode and counter electrode, respectively. In the flow cell test, the gas diffusion electrode of the flow cell, an Ag/AgCl electrode (filled with saturated KCl solution), and foam nickel were used as the cathode (for CO_2_ electroreduction), the reference electrode, and the anode (for oxygen evolution), respectively. Electrochemical data were recorded by an Autolab electrochemical workstation. Before each experiment, high-purity CO_2_ (99.999%) was continuously injected into the cathode electrolyte for 30 min, so that the electrolyte reached saturation. During the electrochemical experiment, CO_2_ was continuously injected at a flow rate of 30 sccm until the end of the experiment. In the electrocatalytic experiment, the cathode electrolyte was stirred with magnetic stirrer (IKA Instrument Equipment Co., Ltd., IKA Guangzhou, China) set at 1000 rpm. The potential (E) reported in this paper is converted to the potential of the reversible hydrogen electrode (RHE) with reference to Formula (1):

E_RHE_ = E_Ag/AgCl_ + 0.197 + 0.0591 × pH (1)

In this study, the pH values of the 0.1 M KHCO_3_ electrolyte saturated with N_2_, Ar, and CO_2_ were 8.6, 8.5, and 6.8, respectively. First, the linear sweep curve (LSV) was performed in CO_2_-saturated or N_2_-saturated 0.1 M KHCO_3_ solution at a scanning rate of 50 mV s^−1^. Then, the ECO_2_RR performance of the catalyst was tested by performing a potentiostatic electrolysis experiment at different potentials, with continuous electrolysis maintained at each potential for 36 min. Before each period of electrolysis, 30 cycles of voltammetry (CV) were used to activate the catalyst.

During the electrochemically active specific surface area (ECSA) test, a 0.1 M KHCO_3_ solution was first saturated with N_2_ for 30 min, and then the CV tests were repeated at different sweep rates (10–60 mV s^−1^) within the potential range of 0.3–0.5 V (vs. RHE, non-Faraday range). At each sweep rate, the number of CV cycles was 20. Electrochemical impedance spectroscopy (EIS) was recorded at an amplitude of 5 mV and frequency of 0.1–10^5^ Hz after the 0.1 M KHCO_3_ solution had been saturated with CO_2_ for 1 h.

### 3.5. Product Analysis

The concentrations of gaseous samples (CO and H_2_) of the electrochemical CO_2_ reduction products were determined by on-line gas chromatography (GC, GC 8860) with a flame ionization detector (FID) and thermal conductivity detector (TCD). During potentiostatic electrolysis, samples were collected every 12 min, and the concentrations of CO and H_2_ they contained were determined by FID and TCD, respectively. The liquid products were quantified by high-performance liquid chromatography (HPLC, HPLC 1200) and methanol was used as the mobile phase.

The Faraday efficiency and partial current density of CO and H_2_ can be calculated by following Equations (2) and (3):

(2) $$FE=\frac{2FvMP}{RTi_{total}}\times 100\%$$

(3) $$FE=\frac{2\times 96485.3\times v\times 10^{-6}\times V_{CO_{2}}\times 10^{-6}\times 101300}{8.314\times 298.15\times i_{total}\times 60}\times 100\%$$

(4) $$j=\frac{\mathrm{FE}\times i_{\mathrm{total}}}{\mathrm{effective}\;\mathrm{active}\;\mathrm{area}}$$

Here, v (ppm) is the volume fraction of the gas-phase products (CO and H_2_); V_CO2_ (mL min^−1^) is the flow rate of CO_2_ (30 mL min^−1^); i_total_ (s) is the total current of the potentiostatic electrolysis; F is Faraday’s constant, 96,485.3 C mol^−1^; P is atmospheric pressure, 101,300 Pa; R is the gas constant, 8.314 J mol^−1^ K^−1^; and T is Kelvin, 298.15 K. The unit of j is mA cm^−2^.

### 3.6. Characterization

The morphology of the samples was analyzed using a transmission electron microscope (TEM, JEOL, JEM-2100F, Tokyo, Japan), and the accelerated voltage used was 200 kV. High-resolution TEM (HRTEM, JEOL, JEM-2100F, Japan) and high-angle annular dark-field scanning transmission electron microscopy (HAADF-STEM, JEOL, Japan) images and elemental mappings were collected by a JEM-2100Plus (JEOL, Japan) field-emission transmission electron microscope. X-ray diffraction (XRD, Rigaku, MERCURY CCD, Tokyo, Japan) patterns were recorded on a Bruker D2 Phaser X-Ray powder Diffractometer with Cu Kα radiation, λ = 0.15406 nm. The Raman spectra of the samples were obtained using a spectrometer (Anton-Paar, Cora 5001, Hannover, Germany) equipped with a 532 and 785 nm laser in the region of 500 to 2500 cm^−1^. Nitrogen sorption–desorption measurements (JW-BK100A, Beijing Jingwei Gaobo Instrument Co., Ltd., Beijing, China) were used to analyze the change in the Brunauer–Emmett–Teller (BET) surface area of the samples. The temperature-programmed desorption (TPD) profiles were obtained by Anton-Paar Chem BET, Germany. CO_2_ adsorption isotherms were determined by a Micromeritics ASAP 2020M (Micromeritics, Norcross, GA, USA) at 25 °C, and before the CO_2_ adsorption experiment, two cycles of gas desorption were performed. X-ray photoelectron spectroscopy (XPS) was recorded on a Thermo Scientific K-Alpha (Thermo Fisher, Waltham, MA, USA). The electron conductivity of the samples was characterized by a four-probe powder resistivity tester (Suzhou Jingge Electronic Co., Ltd., Suzhou, China).

## 4. Conclusions

In summary, we systematically investigated the ECO_2_RR performance of NCBx catalysts with varying nitrogen configurations. Among the tested catalysts, NCB3 demonstrated the optimal performance, achieving high CO selectivity (64.14% in an H-type cell) and activity (partial current densities of 1.9 mA cm^−2^ for CO), along with superior H_2_ selectivity and activity. Importantly, NCB3 produced syngas with adjustable CO/H_2_ ratios (CO/H_2_ = 0.52–4.77) over a wide range of applied potentials (−0.5 to −1.0 V), aligning with the ideal syngas ratio for practical applications. Furthermore, stability tests confirmed the excellent durability of NCB3, which maintained consistent activity and selectivity during extended electrolysis. This work highlights the importance of achieving precise control over nitrogen doping to engineer catalysts with high activity, selectivity, and tunable product distributions for ECO_2_RR. Our findings provide a robust strategy for designing advanced carbon-based catalysts for syngas production and open new avenues for efficient and sustainable CO_2_ utilization in renewable energy systems.

## Data Availability

All data generated or analyzed during this study are included in the published article and its Supplementary Materials.

## Supplementary Materials

The following supporting information can be downloaded at https://www.mdpi.com/article/10.3390/molecules30040953/s1. Figure S1: (a), (b), (c) and (d) Pictures of p-NCB1, p-NCB2, p-NCB3 and p-NCB4. (e), (f), (g) and (h) TEM images of NCB1, NCB2, NCB3 and NCB4. Figure S2: The high-resolution C 1s XPS spectra of NCB1 (a), NCB2 (b), NCB3 (c) and NCB4 (d). Figure S3: (a), (b), (c) and (d) The high-resolution O 1s XPS spectra of NCB1, NCB2, NCB3 and NCB4. Figure S4: NH_3_-TPD of CB, NCB1, NCB2, NCB3 and NCB4. Figure S5: Contact angle of (a) NCB1, (b) NCB2, (c) NCB3 and (d) NCB4. Figure S6: The physical picture of H-type cell and flow cell. Figure S7: LSV curves of NCB1, NCB2, NCB3, NCB4 and CB in CO_2_-saturated 0.1 M KHCO_3_ solution. Figure S8: Total current density of CB, NCB1, NCB2, NCB3 and NCB4 at different electrolytic potentials in H-type cell. Figure S9: Faradaic efficiency of H_2_ and CO in a H-type cell on the various catalysts at different potentials in CO_2_-satureted 0.1 M KHCO_3_ solution. (a) NCB1, (b) NCB2, (c) NCB3 and (d) NCB4. Figure S10: (a) CO and (b) H_2_ FE of NCB1, NCB2, NCB3, NCB4 and CB. Figure S11: (a) FE_CO_ and (b) j_CO_ for NCB3 when alternatively, supplying CO_2_ and Ar feedstocks at -0.9 V vs. RHE. (c) FE_H2_ and (d) j_H2_ for NCB3 when alternatively supplying CO_2_ and Ar feedstocks at -0.9 V vs. RHE. Figure S12: The ratio of syngas at different potentials in H-type cell. Figure S13: CV curves of various catalysts studied in 0.1 M KHCO_3_ at different scan rates (10, 20, 30,40, 50 and 60 mV s^−1^) for estimation of double layer capacitance. (a) NCB1, (b) NCB2, (c) NCB3 and (d) NCB4. Figure S14: The relationship between amino-N (a) and pyridinic-N (b) content of NCB3 and FECO and FE_H2_ in a H-type cell and flow cell, respectively. Figure S15: The relationship between the content of pyrrolic-N (a-b), graphitic-N (c-d), and oxide-N (e-f) and FE_CO_ and FE_H2_ in H-type cell and flow cell, respectively. Table S1: Weight of various sample before and after calcination. Table S2: C, N and O contents of various samples (data from XPS analysis). Table S3: C-C/C=C, C-O, C-N and C=O groups contents of various samples (data from XPS analysis). Table S4: C=O and C-O groups contents of various samples (data from XPS analysis). Table S5: Pyridinic-N, amino-N, pyrrolic-N, graphitic-N and oxide-N contents of various samples. Table S6: The results of N2 adsorption-desorption for different samples. Table S7: Performance comparison of catalysts for syngas formation. Table S8: Electrical conductivity of the synthesized catalysts by the 4-point probe method under a pressure of 10 MPa.
