# Supplementary material for: Rational Design of Metal-Free Nitrogen-Doped Carbon for Controllable Reduction of CO2 to Syngas
Source: Molecules. 2025 Feb 18;30(4):953. doi: 10.3390/molecules30040953 (PMC11858690; doi:10.3390/molecules30040953)
Supplement: Supplementary file 1 [file molecules-30-00953-s001.zip › molecules-3482904-supplementary.pdf]

# Rational Design of Metal-Free Nitrogen-Doped Carbon for Controllable Reduction of $CO_2$ to Syngas

**Guangbin An [1], Kang Wang [1], Min Yang [2], Jiye Zhang [3], Haijian Zhong [2,*], Liang Wang [1] and Huazhang Guo [1,*]**

[1] Institute of Nanochemistry and Nanobiology, School of Environmental and Chemical Engineering, Shanghai University, 99 Shangda Road, Shanghai 200444, China
[2] School of Information Engineering, Gannan Medical University, Ganzhou 341000, China
[3] School of Materials Science and Engineering, Shanghai University, 99 Shangda Road, Shanghai 200444, China
* Correspondence: hjzhong2007@gmu.edu.cn (H.Z.); guohuazhang@shu.edu.cn (H.G.)

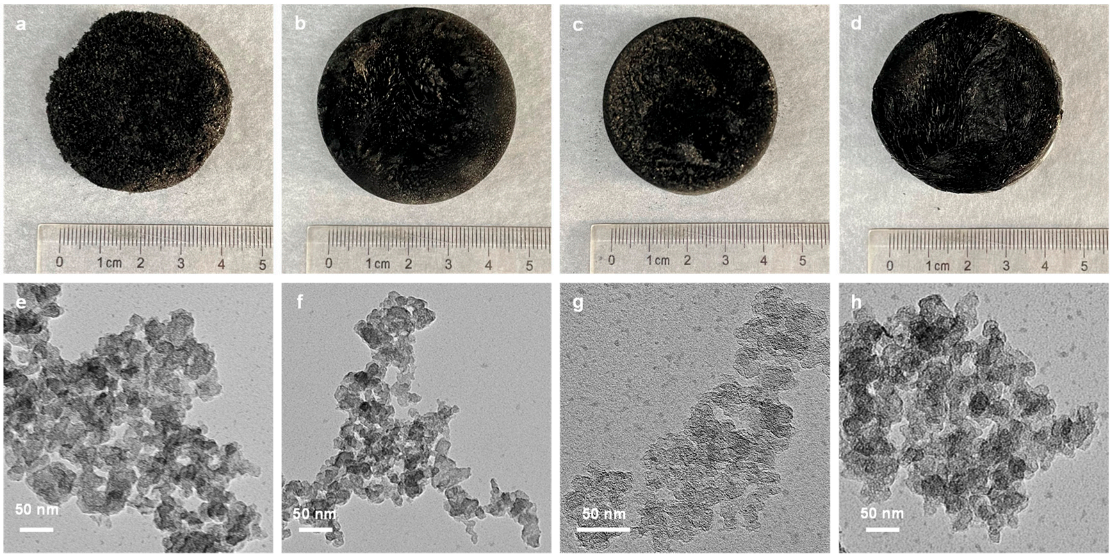


**Figure S1.** (**a**), (**b**), (**c**) and (**d**) Pictures of p-NCB1, p-NCB2, p-NCB3 and p-NCB4. (**e**), (**f**), (**g**) and (**h**) TEM images of NCB1, NCB2, NCB3 and NCB4.

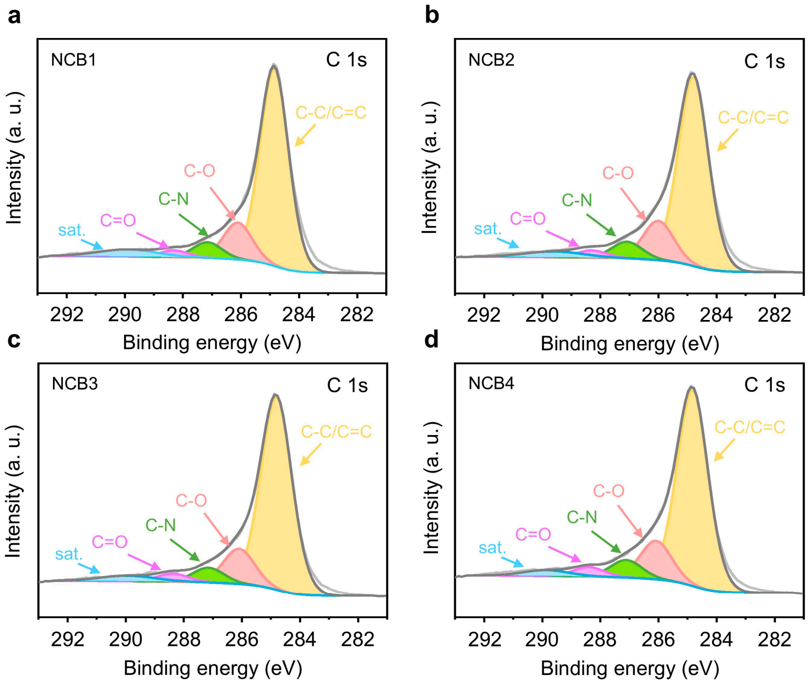


**Figure S2.** The high-resolution C 1s XPS spectra of NCB1 (**a**), NCB2 (**b**), NCB3 (**c**) and NCB4 (**d**).

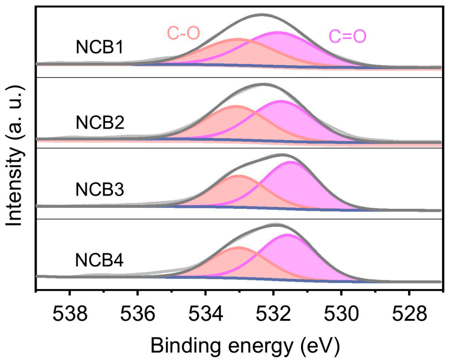


**Figure S3.** (**a**), (**b**), (**c**) and (**d**) The high-resolution O 1s XPS spectra of NCB1, NCB2, NCB3 and NCB4.

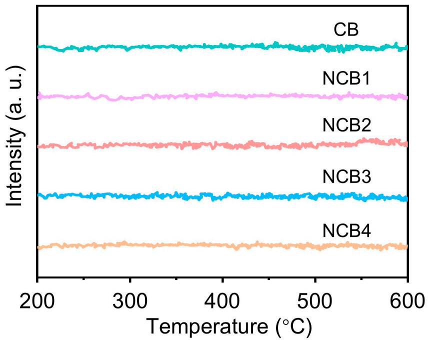


**Figure S4.** $NH_3$-TPD of CB, NCB1, NCB2, NCB3 and NCB4.

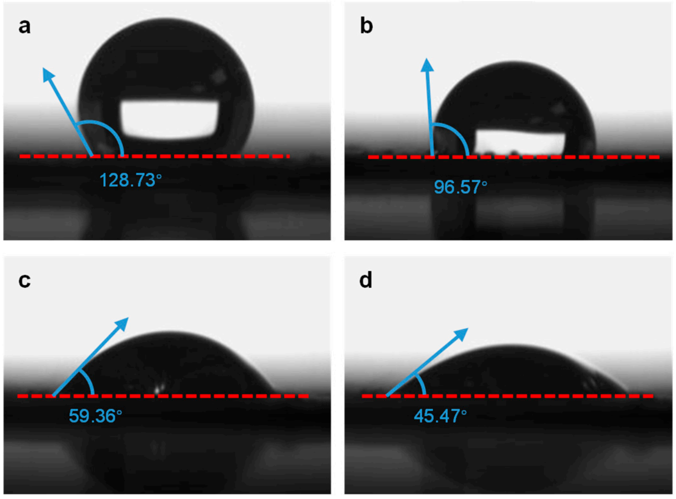


**Figure S5.** Contact angle of (**a**) NCB1, (b) NCB2, (c) NCB3 and (d) NCB4.

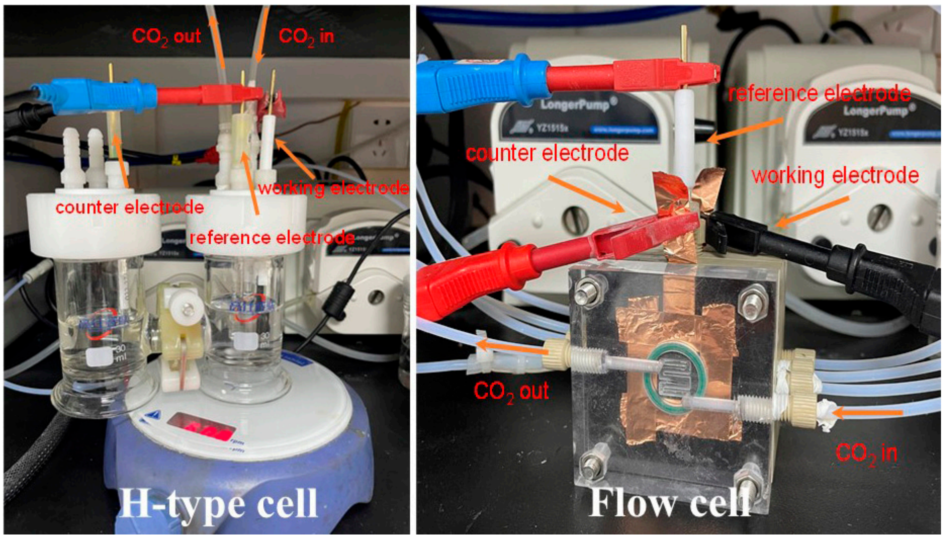


**Figure S6.** The physical picture of H-type cell and flow cell.

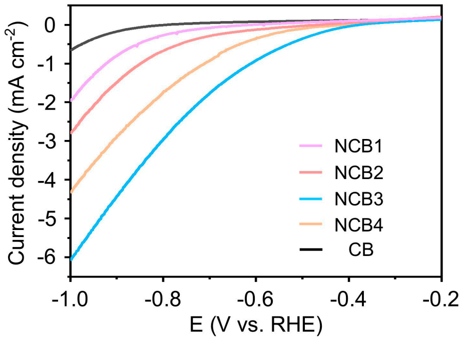


**Figure S7.** LSV curves of NCB1, NCB2, NCB3, NCB4 and CB in $CO_2$-saturated 0.1 M $KHCO_3$ solution.

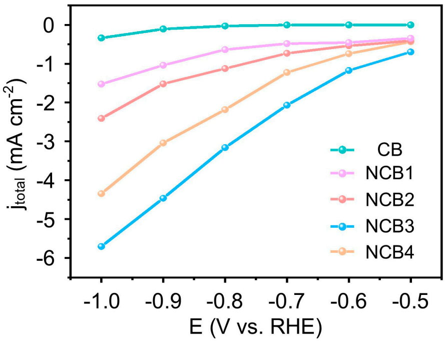


**Figure S8.** Total current density of CB, NCB1, NCB2, NCB3 and NCB4 at different electrolytic potentials in H-type cell.

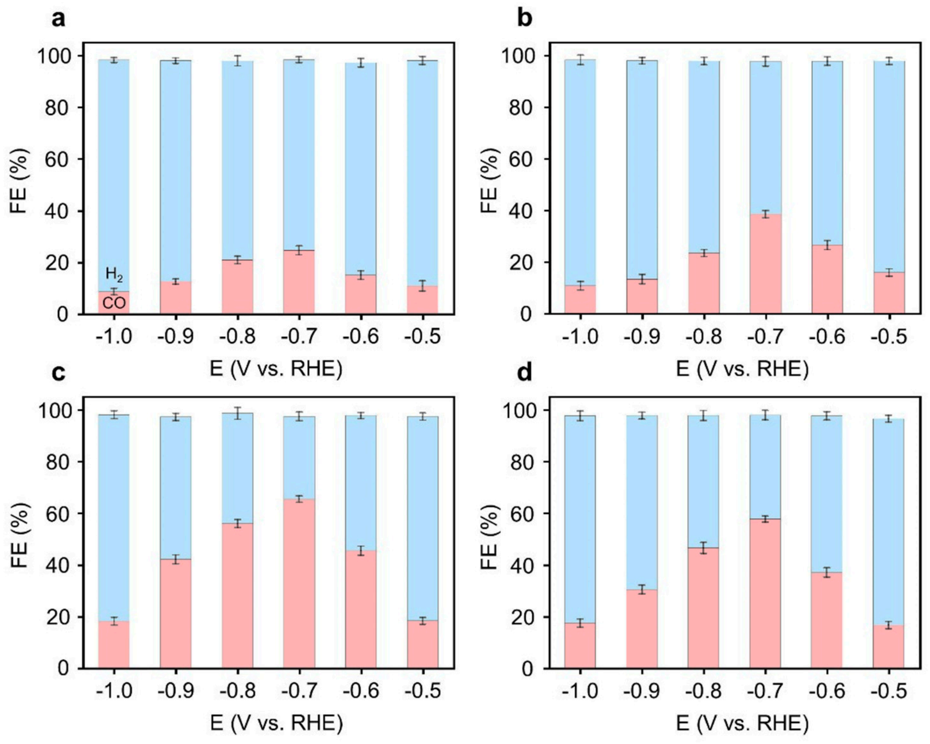


**Figure S9.** Faradaic efficiency of $H_2$ and CO in a H-type cell on the various catalysts at different potentials in $CO_2$-satureted 0.1 M $KHCO_3$ solution. (**a**) NCB1, (**b**) NCB2, (**c**) NCB3 and (**d**) NCB4.

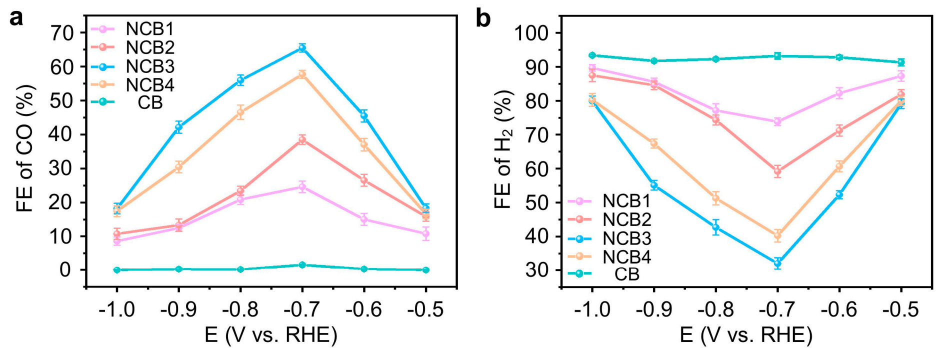


**Figure S10.** (**a**) CO and (b) $H_2$ FE of NCB1, NCB2, NCB3, NCB4 and CB.

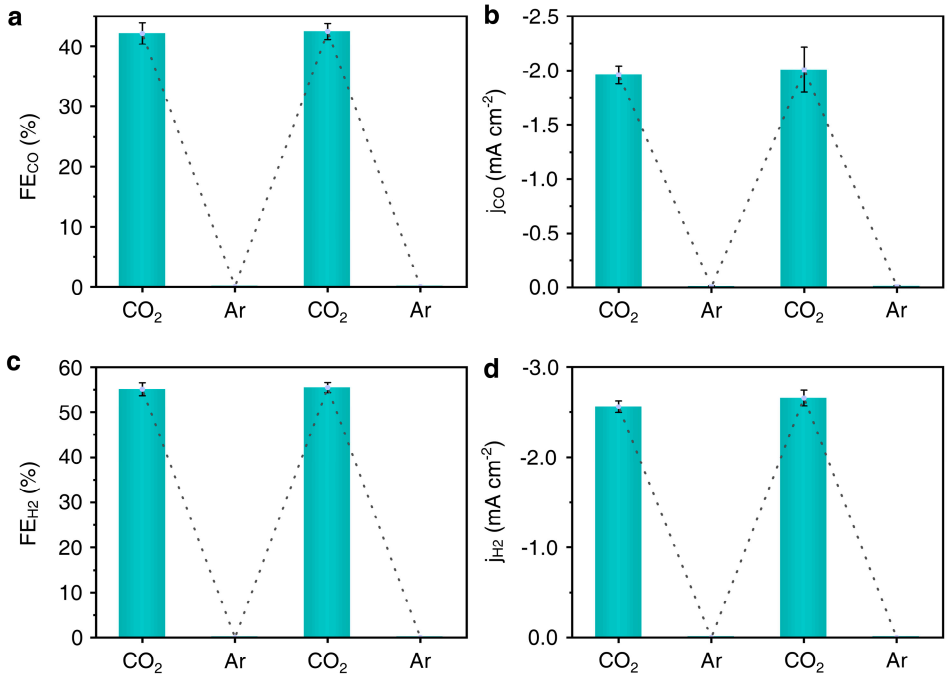


**Figure S11.** (**a**) $FE_{CO}$ and (**b**) $j_{CO}$ for NCB3 when alternatively, supplying $CO_2$ and Ar feedstocks at -0.9 V vs. RHE. (**c**) $FE_{H2}$ and (**d**) $j_{H2}$ for NCB3 when alternatively supplying $CO_2$ and Ar feedstocks at -0.9 V vs. RHE.

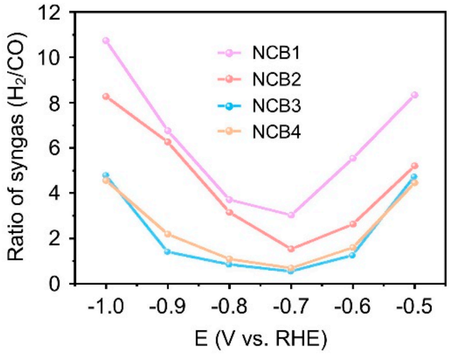


**Figure S12.** The ratio of syngas at different potentials in H-type cell.

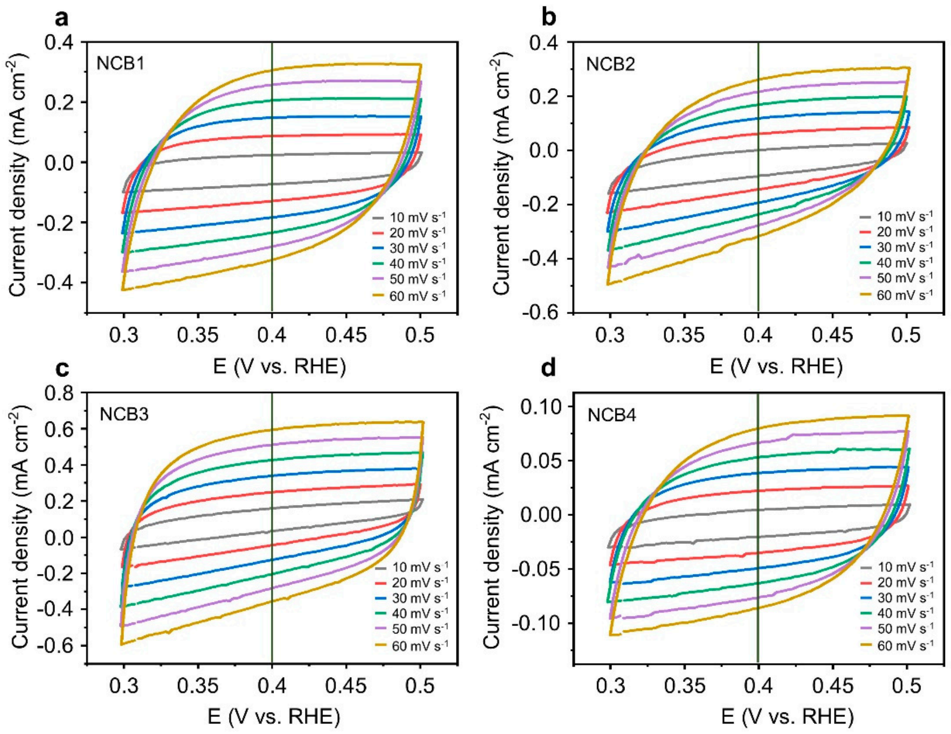


**Figure S13.** CV curves of various catalysts studied in 0.1 M $KHCO_3$ at different scan rates (10, 20, 30,40, 50 and 60 mV $s^{-1}$) for estimation of double layer capacitance. (**a**) NCB1, (**b**) NCB2, (**c**) NCB3 and (**d**) NCB4.

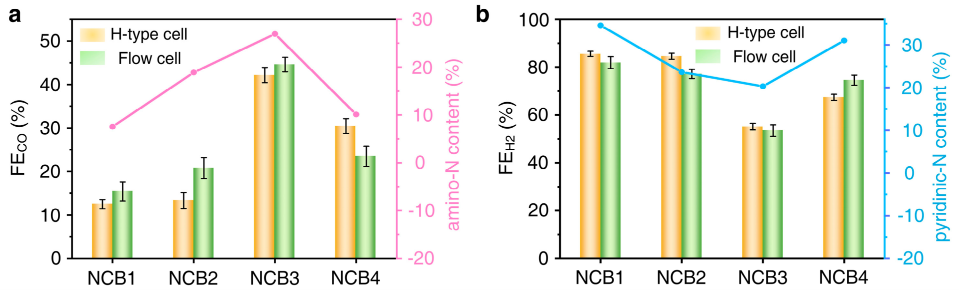


**Figure S14.** The relationship between amino-N (a) and pyridinic-N (b) content of NCB3 and $FE_{CO}$ and $FE_{H2}$ in a H-type cell and flow cell, respectively.

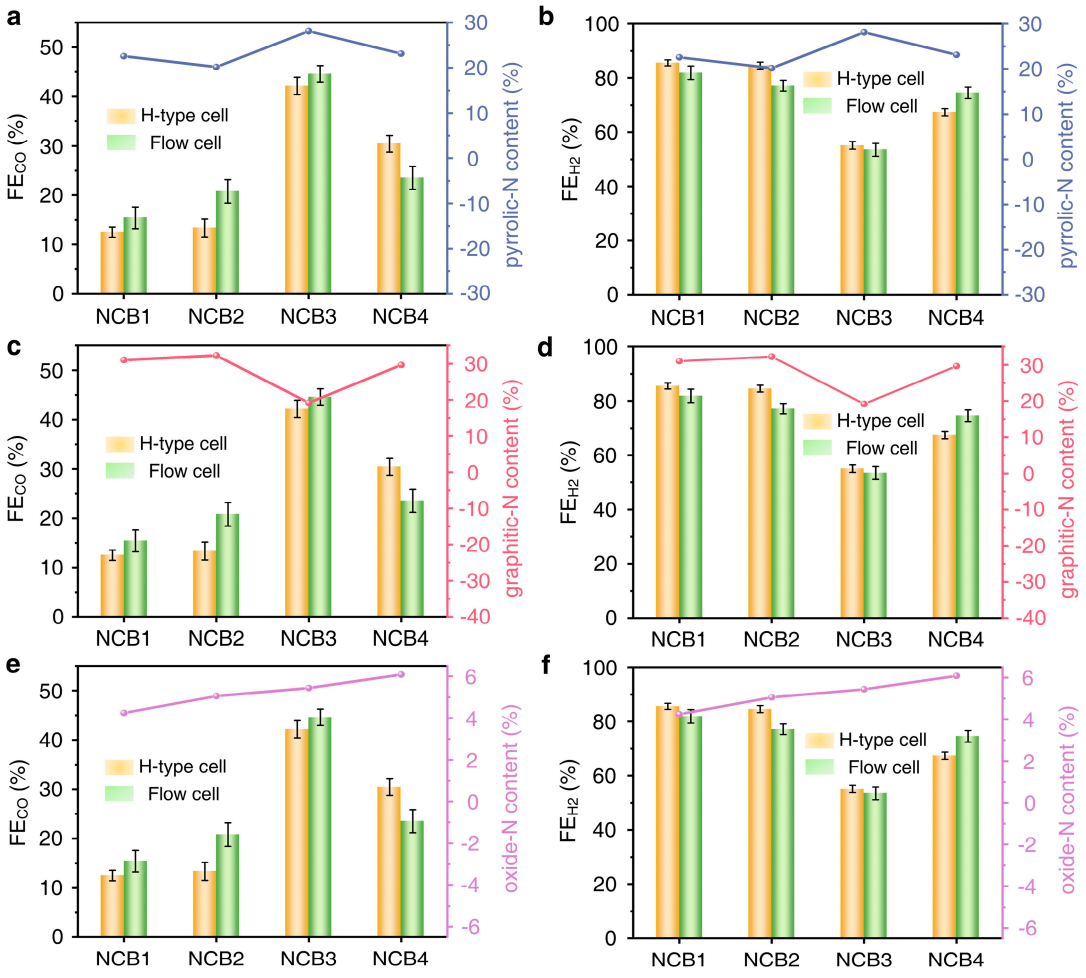


**Figure S15.** The relationship between the content of pyrrolic-N (a-b), graphitic-N (c-d), and oxide-N (e-f) and $FE_{CO}$ and $FE_{H2}$ in H-type cell and flow cell, respectively.

**Table S1.** Weight of various sample before and after calcination.

| Samples | Before calcination (g) | After calcination (g) | Yield (%) |
|---|---|---|---|
| NCB1 | 0.285 | 0.152 | 53.33 |
| NCB2 | 0.488 | 0.203 | 41.60 |
| NCB3 | 0.715 | 0.532 | 74.41 |
| NCB4 | 0.885 | 0.619 | 69.94 |

**Table S2.** C, N and O contents of various samples (data from XPS analysis).

| Samples | C (at.%) | N (at.%) | O (at.%) |
|---|---|---|---|
| CB | 97.04 | - | 2.96 |
| NCB1 | 91.21 | 3.14 | 5.65 |
| NCB2 | 92.46 | 3.18 | 4.36 |
| NCB3 | 90.99 | 4.28 | 4.73 |
| NCB4 | 89.05 | 5.28 | 5.67 |

**Table S3.** C-C/C=C, C-O, C-N and C=O groups contents of various samples (data from XPS analysis).

| Samples | C-C/C=C (at.%) | C-O (at.%) | C-N (at.%) | C=O (at.%) |
|---|---|---|---|---|
| NCB1 | 76.89 | 14.34 | 6.14 | 2.63 |
| NCB2 | 74.83 | 15.58 | 6.68 | 2.91 |
| NCB3 | 76.78 | 14.04 | 5.92 | 3.26 |
| NCB4 | 74.88 | 14.74 | 6.73 | 3.62 |

**Table S4.** C=O and C-O groups contents of various samples (data from XPS analysis).

| Samples | C=O (at.%) | C-O (at.%) |
|---|---|---|
| NCB1 | 56.59 | 43.41 |
| NCB2 | 54.40 | 45.60 |
| NCB3 | 59.88 | 40.12 |
| NCB4 | 59.70 | 40.30 |

**Table S5.** Pyridinic-N, amino-N, pyrrolic-N, graphitic-N and oxide-N contents of various samples.

| Samples | pyridinic-N (at.%) | amino-N (at.%) | pyrrolic-N (at.%) | graphitic-N (at.%) | oxide-N (at.%) |
|---|---|---|---|---|---|
| NCB1 | 34.58 | 7.55 | 22.58 | 31.04 | 4.25 |
| NCB2 | 23.63 | 18.88 | 20.17 | 32.26 | 5.06 |
| NCB3 | 20.30 | 26.94 | 28.12 | 19.20 | 5.44 |
| NCB4 | 31.02 | 10.11 | 23.12 | 29.66 | 6.09 |

**Table S6.** The results of $N_2$ adsorption-desorption for different samples.

| Samples | $S_{BET}$ ($m^2$ $g^{-1}$) [a] | $V_{Total}$ ($cm^3$ $g^{-1}$) [b] | $\bar{D}$ (nm) [c] |
|---|---|---|---|
| CB | 138.24 | 0.082 | 2.37 |
| NCB1 | 268.94 | 0.24 | 3.57 |
| NCB2 | 458.29 | 0.66 | 5.76 |
| NCB3 | 602.71 | 0.86 | 5.71 |
| NCB4 | 582.70 | 0.80 | 5.49 |

[a] $S_{BET}$ (BET surface area) is calculated by BET method. [b] $V_{Total}$ (Total pore volume) is the single point adsorption at $P/P_0$=0.99. [c] $\bar{D}$ (average pore diameter) is calculated by BJH method.

**Table S7.** Performance comparison of catalysts for syngas formation.

| Catalysts | $FE_{CO}$ (%) | $CO/H_2$ ratios | current density ($mA/cm^2$) | applied potentials ($V_{RHE}$) | Ref. |
|---|---|---|---|---|---|
| NCB3 | 64.14 | 0.52-4.77 | 1.9 | -0.5-1.0 V | This work |
| OA-PCN | 40 | 0.67 | -7 | -0.56-0.86 V | 1 |
| Pd/C | 62 | 0.5 | 1.5 | -0.5-0.8 V | 2 |
| MPC-1000 | 62 | not reported | -6 | -0.3-0.7 V | 3 |
| Pd/PdOx | 58 | 1.45 | 11 | 0.55-0.9 V | 4 |
| $Cu_{14}Zn_{86}O$/CNT | not reported | 1 | 1.5 | -0.5-0.88 V | 5 |
| Ni-NG | 53 | 1.10 | 12.4 | -0.5-0.9 V | 6 |
| Co@CoNC-900 | 42 | 1 | -2.7-7.9 | -0.6 -0.8 V | 7 |

**Table S8.** Electrical conductivity of the synthesized catalysts by the 4-point probe method under a pressure of 10 MPa.

| Samples | NCB1 | NCB2 | NCB3 | NCB4 |
|---|---|---|---|---|
| electrical resistivity (mΩ cm) | 239.5 | 149.3 | 70.1 | 84.3 |
| conductivity $\times 10^3$ (S $cm^{-1}$) | 4.2 | 6.7 | 14.3 | 11.8 |

## References


1. Meng, N.; Zhou, W.; Yu, Y.; Liu Y.; Zhang, B. Superficial hydroxyl and amino groups synergistically active polymeric carbon nitride for $CO_2$ electro-reduction. *ACS Catal.* **2019**, *9*, 10983-10989.
2. Sheng, W.; Kattel, S.; Yao, S.; Yan, B.; Liang, Z.; Hawxhurst, C.; Wu, Q.; Chen, J. G. Electrochemical reduction of $CO_2$ to synthesis gas with controlled $CO/H_2$ ratios. *Energy Environ. Sci.* **2017**. *10*, 1180-1185.
3. Pan, F.; Liang, A.; Duan, Y.; Liu, Q.; Zhang J.; Li, Y. Self-growth templating synthesis of 3D N,P, co-doped mesoporous carbon frameworks for efficient bifunctional oxygen and carbon dioxide electroreduction. *J. Mater. Chem. A* **2017**, *5*, 13104-13111.
4. Lu, H.; Zhang, L.; Zhong, J. H.; Yang, H.G. Partially oxidized palladium nanodots for enhanced electrocatalytic carbon dioxide reduction. *Chem.-Asian J.* **2018**, *13*, 2800-2804.
5. Hjorth, I.; Nord, M.; Rønning, M.; Yang, J.; Chen, D. Electrochemical reduction of $CO_2$ to synthesis gas on CNT supported $Cu_xZn_{1-x}O$ Catalysts. *Catal. Today* **2020**, 357, 311-321.

6. Jiang, K.; Siahrostami, S.; Zheng, T.; Hu, Y.; Hwang, S.; Stavitski, E.; Peng, Y.; Dynes, J.; Gangisetty, M.; Su, D.; Attenkofer, K.; Wang. H. Isolated Ni single atoms in graphene nanosheets for high-performance $CO_2$ reduction. *Energy Environ. Sci.* **2018**, *11*, 893-903.
7. Daiyan, R.; Chen, R.; Kumar, P.; Bedford, N.M.; Qu, J.; Cairney, J.M.; Lu, X.; Amal. R. Tuneable syngas production through $CO_2$ electroreduction on cobalt-carbon composite electrocatalyst. *ACS Appl. Mater. Interfaces* **2020**, *12*, 9307-9315.
